# Tickling the heart: integrating social emotional learning into medical education to cultivate empathetic, resilient, and holistically developed physicians

**DOI:** 10.3389/fmed.2024.1368858

**Published:** 2024-03-04

**Authors:** Wei-Chin Hsu, Lih-Jyh Fuh, Shih-Chieh Liao

**Affiliations:** ^1^School of Dentistry, China Medical University, Taichung, Taiwan; ^2^Dental Department, China Medical University Hospital, Taichung, Taiwan; ^3^Medical School, China Medical University, Taichung, Taiwan

**Keywords:** social-emotional learning (SEL), medical education, core competences, empathy, resilience

## Abstract

**Objectives:**

Advancements in technology have improved healthcare quality but shifted the focus to efficiency, negatively impacting patient– doctor relationships. This study proposes integrating social-emotional learning (SEL) into medical education to address this issue.

**Key arguments:**

Social-emotional learning (SEL) is based on social learning theory and has a focus on emotion management, stress management, empathy, and social skills. Through SEL, students can develop social and emotional skills by observing, interacting with, and imitating others. Incorporating SEL into medical education would ensure that physicians develop the social and emotional skills necessary to form positive relationships with patients and to cope with the emotional demands of medical work. SEL comprises six domains, namely, the cognitive, emotion, social, values, perspective, and identity domains. These six domains are closely related to the six core competencies the Accreditation Council for Graduate Medical Education (ACGME) indicated every doctor should possess, which indicates that the domains of SEL are highly relevant within the context of medical education. Furthermore, SEL can lead to the development of empathy, which can improve physicians’ ability to understand patients’ perspectives and emotions, and resilience, which can enable physicians to more effectively cope with the demands of their work, and it can lead to holistic development, with doctors gaining an understanding of both the technical and humanistic aspects of their work.

**Conclusion:**

Incorporating SEL in medical education would enable doctors to develop key social and emotional skills that would improve their ability to provide holistic medical services and therefore would improve overall medical systems.

## Background

Although developments in medical technology, including those involving the application of artificial intelligence, have improved the quality of healthcare, they may have led to physicians forming weaker interpersonal relationships with patients (1). When physicians focus too strongly on the technology-based aspects of their work, they may forget the value of humanity and the patients’ sense of self-worth and may consequently overlook the emotional needs of their patients, compromising the quality of the doctor–patient relationship (2). Therefore, the pursuit of technological advancement in medicine without adequate consideration to educating and training physicians in ethics and the emotional aspects of their work may result in physicians who are unable to properly reflect on and consider patients’ needs and values when engaged in ethical decision-making.

Emotions, emotion management, and emotional intelligence play crucial roles in the work of healthcare professionals (3). Healthcare professionals often face highly stressful and emotionally demanding situations at work, and frequent exposure to such situations can have negative effects on their mental health and work performance (4). Ensuring that healthcare professionals have experience with and are equipped with strategies for emotion management is crucial to improving their overall wellbeing and quality of healthcare. Mindfulness and emotional intelligence, which are closely related concepts, have protective effects on healthcare professionals. Therefore, cultivating mindfulness, emotional intelligence, and emotion management skills in healthcare workers can help them to more effectively cope with work-related stress, improve their emotion management skills, and improve their overall work quality (3, 4).

Healthcare is an interactive process, and focusing on technological advancements without considering the humanity and self-awareness of physicians can lead to ineffective patient-physician relationships and low healthcare quality. In addition to improving physicians’ understanding of technological advancements, medical educators should cultivate physicians’ awareness of the value of humanity and self-worth as well as their mindfulness, emotional intelligence, emotion management, and ability to self-reflect (1–4). Such an education would enable healthcare providers to strike a balance between applying their expertise and exercising their humanity, enabling the provision of comprehensive and compassionate medical care.

### Social-emotional learning

Social-emotional learning (SEL) was proposed by the Collaborative for Academic, Social, and Emotional Learning (CASEL) in the United States. It is a form of emotional education that fosters understanding one’s own and others’ emotions, managing stress, exercising empathy, and developing social skills (5–7). SEL is based on social learning theory, which emphasizes that individuals learn behaviors and skills through observation, imitation, and social interaction. SEL focuses on the cultivation of social and emotional abilities among students, including those related to emotion management, interpersonal relationships, conflict resolution, and value development.

The development and implementation of SEL curricula typically draw upon the principles of social learning theory (8, 9). In educational programs based on SEL, students acquire knowledge through the observation and emulation of the social and emotional behaviors of peers and by actively participating in social interactions. This method of learning aligns with the core principles of social learning theory, which posits that learning is a social process, and that individuals acquire knowledge and skills through their interactions with others (8, 9). The development of SEL expanded the scope of application of social learning theory because in addition to focusing on how students learn social behaviors, it considers how educators can cultivate emotional intelligence and abilities among students. Such cultivation can help students recognize, understand, and effectively manage their own emotions, which can lead to their personal growth and wellbeing. In summary, SEL, with the principles of social learning theory at the core, can be used to develop social and emotional competencies among students. Structured curricula and activities based on SEL can cultivate crucial skills among students at all levels of education, including those related to emotion management, interpersonal relationships, and value development, which can improve the likelihood of growth and success.

### Six domains of SEL

Social-emotional learning consists of primary core competencies in six domains: cognitive domain, emotion domain, social domain, value domain, perspective domain, and identity domain (5, 6). These competencies encompass several skills and abilities that are essential to students’ social and emotional development.

The cognitive domain focuses on the fundamental cognitive skills required by students to achieve goals. Such skills include attention control, management of working memory and planning, inhibition control, cognitive flexibility, and critical thinking.

The emotion domain encompasses the skills that help individuals recognize, express, and regulate their emotions as well as understand and empathize with others. Developing the skills in this domain is crucial to managing one’s own emotions and behaviors and engaging in positive interactions with others. These skills include emotional understanding and expression, emotional and behavioral regulation, empathy, and perspective taking.

The social domain involves skills that help an individual accurately interpret others’ behavior, effectively navigate social situations, and effectively engage in positive interactions with others. Developing the skills in this domain is crucial to participating in collaborative work, solving social problems, building positive relationships, and living harmoniously with others, and such skills involve understanding social cues, implementing conflict resolution and social problem-solving, and employing prosocial and cooperative behaviors.

The value domain includes skills, character traits and virtues, and practices that enable individuals to become prosocial and productive members of their communities. This domain involves understanding, caring, and actions related to core moral values; a desire to achieve one’s potential; and the habits required to live and work with friends, family members, and community members. The values encompassed in this domain include moral, performance, intellectual, and civic values.

The perspective domain involves a person’s outlook on and approach to interacting with the world. It influences how individuals perceive themselves, others, and their circumstances, and it shapes how they interpret and respond to challenges in their daily lives. Having a positive perspective can prevent the development of and facilitate the management of negative emotions and can enable an individual to successfully complete tasks and interact harmoniously with others. A positive perspective involves being optimistic, grateful, open, or enthusiastic.

The identity domain encompasses an individual’s understanding and perception of themselves and their capabilities, including their knowledge and beliefs about themselves and their ability to learn and grow. When individuals are satisfied with themselves; have a secure sense of their place in the world; and are confident in their ability to learn, grow, and overcome obstacles, they are better equipped to face challenges and build positive relationships. This domain includes self-awareness, goal-setting, self-efficacy and a growth mindset, and self-esteem.

## Connections between the six domains of SEL and six core competencies of the Accreditation Council for Graduate Medical Education

The Accreditation Council for Graduate Medical Education (ACGME) recommended that physicians should have six core competencies (i.e., medical knowledge, professionalism, interpersonal and communication skills, systems-based practice, practice-based learning and improvement, and patient care), (10) which are closely aligned with the six domains of the SEL framework.

### Cognitive domain versus medical knowledge

The cognitive domain of SEL, which focuses on attention control, management of working memory, critical thinking, and cognitive flexibility, is closely related to the ACGME core competency of medical knowledge. By developing strong cognitive skills, healthcare professionals can acquire, absorb, and apply medical knowledge, which can improve their diagnostic accuracy and evidence-based decision-making and ensure effective patient care.

### Emotion domain versus professionalism

The emotion domain of SEL encompasses emotional knowledge and expression, emotion regulation, and empathy and therefore closely aligns with the ACGME core competency of professionalism. Emotional intelligence and empathy enable healthcare professionals to establish deeper connections with patients, demonstrate compassion and respect, and ensure the ethicality of their behavior. These skills help them build trust, develop positive physician–patient relationships, and provide patient-centered care.

### Social domain versus interpersonal and communication skills

The social domain of SEL, which involves understanding social cues, conflict resolution, and normative behavior, aligns with the ACGME core competency of interpersonal and communication skills. Effective communication, collaboration, and teamwork are essential for interactions between healthcare professionals and patients, patients’ families, and interdisciplinary teams. By developing social skills, physicians can navigate complex social dynamics, establish positive relationships, resolve conflicts, and promote a harmonious healthcare environment.

### Value domain versus systems-based practice

The value domain of SEL, which encompasses moral values, performance values, intellectual values, and civic values, aligns with the ACGME core competency of systems-based practice. The strong values, ethics, and moral principles of healthcare professionals guide their practice and enable them to prioritize patient wellbeing and account for the effects of their healthcare decisions on individuals and communities. Strong values can lead physicians to engage in system thinking, advocate for patient rights, and participate in community health initiatives.

### Perspective domain versus practice-based learning and improvement

The SEL perspective domain, which emphasizes optimism, gratitude, openness, and enthusiasm, aligns with the ACGME core competency of practice-based learning and improvement. Having a positive perspective can help physicians navigate challenges, manage stress, and engage in lifelong learning. In addition, such a perspective leads to a growth mindset, encourages reflection, and fosters resilience, all of which promote ongoing professional development and excellence in medical practice.

### Identity domain versus patient care

The SEL identity domain, which involves self-awareness, goal-setting, self-efficacy, and self-esteem, is related to the ACGME core competency of personal and professional development. By establishing a strong professional identity, healthcare professionals can gain a deep understanding of their values, strengths, and motivations. This self-awareness supports personal growth, career planning, and the cultivation of self-efficacy. It also leads to confidence, resilience, and general wellbeing, which can help physicians navigate challenges and maintain their professional competence. The relationship between the six SEL domains and the six ACGME competences can be found in Supplementary Table 1.

Because of the relevance of the SEL domains to the ACGME core competencies, the SEL framework should be integrated into medical education and training programs for the development of the ACGME core competencies among healthcare professionals. By utilizing SEL, educators can cultivate well-rounded physicians who possess both technical expertise and strong social and emotional skills. This integration can lead to the development of compassionate, and resilient healthcare professionals who provide high-quality care, improving patient wellbeing and the overall healthcare system.

## Implications of SEL in medical education

The abilities specified in SEL develop at different life stages. During early childhood, people generally develop emotion management and basic social skills, including the abilities to recognize and express emotions, share, and cooperate. In childhood and adolescence, more complex skills are developed, such as problem-solving, self-awareness, empathy, and coping abilities. In adulthood, self-planning skills, emotional intelligence, the ability to form interpersonal relationships, and skills related to career development are developed (11).

### Empathy

Medical education focused on SEL principles can cultivate empathy in medical students through empathy training. Students can learn how to understand and empathize with patients’ perspectives and emotions and how to respond appropriately to their needs. Furthermore, such education can help medical students establish trust and feel empathic resonance with patients, providing more personalized healthcare services with greater compassion.

### Resilience

Medical education based on SEL holds promise for fostering resilience among medical students. Firstly, SEL education aids students in gaining a deeper comprehension of emotions and their management, thereby enabling them to effectively confront stressors and challenges with reduced susceptibility to emotional fluctuations. Secondly, through SEL education, students acquire techniques and strategies for coping with stress, a pivotal skillset for addressing challenges within the demanding milieu of healthcare environments. Furthermore, SEL education promotes students’ understanding of patients’ needs and emotional states, concurrently fostering the development of peer support networks, which can provide invaluable psychological support during times of adversity. Most significantly, this form of education extends beyond the purview of medical knowledge and technical skills, emphasizing ethical, humanistic, and moral dimensions, thereby enabling students to adopt a more comprehensive approach when confronted with diverse situations. SEL education contributes significantly to the cultivation of resilience in medical students, equipping them with enhanced capabilities to effectively navigate the multifaceted challenges inherent to the medical profession.

### Holistic development

The focus of SEL-based medical education is holistic development, and medical students are encouraged to practice comprehensive growth. In addition to learning medical knowledge and skills, students cultivate moral judgment, professional ethics, teamwork skills, and lifelong learning abilities. Such education equips medical students with various competencies because it focuses on both the technical and the ethical and humanistic aspects of medical services, enabling healthcare professionals to provide comprehensive healthcare services.

In conclusion, the integration of SEL principles into medical education carries three significant implications for the training and development of healthcare professionals. Firstly, by emphasizing SEL, medical educators can contribute to the cultivation of compassionate, empathetic, and resilient practitioners who provide high-quality care and positively influence the wellbeing of their patients. These educational initiatives equip prospective healthcare professionals with self-regulation skills, emotional intelligence, the ability to establish interpersonal relationships, and the essential competencies for professional growth. Secondly, the integration of SEL into medical education ensures that physicians possess both the necessary technical knowledge and social-emotional skills, thereby facilitating their professional advancement and enhancing the overall wellbeing and healthcare quality of their patients.

Thirdly, the infusion of SEL principles into medical education can foster the development of empathy and resilience among future physicians, thereby contributing to their holistic growth. In today’s technological era, there has been a predominant focus on efficiency (1, 2). However, medical education enriched with SEL places a greater emphasis on humanistic care. Consequently, physicians educated in this manner provide more comprehensive and superior-quality healthcare services.

This study has explored the incorporation of SEL within medical education, with its primary objective being to provide medical educators and administrators with a foundational understanding of SEL principles. The development of SEL competencies among prospective physicians can ensure positive interpersonal relationships and proficiency in emotional management. Moreover, these individuals can serve as role models and inspire others within the organization to engage in SEL practices (12). This study represents an initial exploration of the application of SEL in medical education, paving the way for future researchers to delve deeper into the relationships between instructional approaches and the enhancement of SEL competencies among medical students.

Future medical educators can ensure the integration of SEL clinical courses into medical curricula and conduct appropriate qualitative course assessments through the following approaches:

1. Social-emotional learning (SEL) course design from basic to clinical learning: Medical educators should design courses based on SEL principles, covering topics such as emotional management, empathy cultivation, and conflict resolution. These courses should span the entire undergraduate medical curriculum, ensuring students receive comprehensive social and emotional learning throughout their medical training. The medical curriculum should be aligned with students’ personal development and societal context, enabling students to integrate learned social and emotional skills across various learning contexts.
2. Incorporation of educational ecological perspectives: Both SEL course (13, 14) and medical education (15) designs emphasize the significance of educational ecology, as interactions between students and all stakeholders in the educational ecosystem influence students’ learning outcomes and development. Educational ecological perspectives should be a crucial consideration in course design to ensure adaptation to the diverse needs and expectations of stakeholders. During course evaluation, the impact of educational ecology should be considered, and course content and teaching methods should be continually adjusted and improved based on feedback.
3. Appropriate qualitative course assessment: Medical educators need to develop effective qualitative assessment methods to evaluate the impact of SEL courses on students. These assessments may include qualitative surveys and observations of students’ emotional intelligence levels, empathy skills, and emotional management abilities. Assessment results should comprehensively consider various factors in the educational environment, including course structure, teaching methods, and learning resources, to evaluate course effectiveness. Medical educators should collect and analyze evaluation data from SEL-based clinical courses to assess their impact on student empathy and holistic approaches. These data can guide further improvements and adjustments to the course. Data collection and analysis should consider students’ individual differences, social backgrounds, and the impact of the course on students’ personal development and societal systems.
4. Continuous development and enhancement of SEL course cycles: Medical educators can facilitate more research to explore the impact of SEL-based clinical courses on student-doctor relationships. Continuous research and development efforts can continually refine and enhance medical education, nurturing doctors with greater empathy and holistic approaches to promote the development of doctor-patient relationships. Additionally, attention should be paid to social-cultural factors and institutional environments to foster the overall development and transformation of medical education.

## Author contributions

W-CH: Writing – original draft, Writing – review & editing, Conceptualization. L-JF: Writing – original draft, Writing – review & editing. S-CL: Writing – original draft, Conceptualization, Funding acquisition, Writing – review & editing.
