# Supplementary material for: Tickling the heart: integrating social emotional learning into medical education to cultivate empathetic, resilient, and holistically developed physicians
Source: Front Med (Lausanne). 2024 Mar 4;11:1368858. doi: 10.3389/fmed.2024.1368858 (PMC10944992; doi:10.3389/fmed.2024.1368858)
Supplement: Supplementary file 1 [file Data_Sheet_1.PDF]

Table 1 Six SEL domains and the six ACGME competences they are relevant to

Six SEL domains	Six ACGME core competencies
Cognitive domain	Medical knowledge
Emotion domain	Professionalism
Social domain	Interpersonal and communication skills
Value domain	Systems-based practice
Perspective domain	Practice-based learning and improvement
Identity domain	Patient care
